# Spinal Loads during Cycling on an Ergometer

**DOI:** 10.1371/journal.pone.0095497

**Published:** 2014-04-17

**Authors:** Antonius Rohlmann, Thomas Zander, Friedmar Graichen, Hendrik Schmidt, Georg Bergmann

**Affiliations:** Julius Wolff Institute, Charitè – Universitätsmedizin Berlin, Berlin, Germany; Delft University of Technology (TUDelft), Netherlands

## Abstract

Cycling on an ergometer is an effective exercise for improving fitness. However, people with back problems or previous spinal surgery are often not aware of whether cycling could be harmful for them. To date, little information exists about spinal loads during cycling. A telemeterized vertebral body replacement allows *in vivo* measurement of implant loads during the activities of daily living. Five patients with a severe compression fracture of a lumbar vertebral body received these implants. During one measurement session, four of the participants exercised on a bicycle ergometer at various power levels. As the power level increased, the maximum resultant force and the difference between the maximum and minimum force (force range) during each pedal revolution increased. The average maximum-force increases between the two power levels 25 and 85 W were 73, 84, 225 and 75 N for the four patients. The corresponding increases in the force range during a pedal revolution were 84, 98, 166 and 101 N. There were large variations in the measured forces between the patients and also within the same patient, especially for high power levels. In two patients, the maximum forces during high-power cycling were higher than the forces during walking measured on the same day. Therefore, the authors conclude that patients with back problems should not cycle at high power levels shortly after surgery as a precaution.

## Introduction

Sport is usually associated with physical activity and is thus an important factor for the prevention and palliation of various diseases. After spinal surgery, the physical performance of patients is often drastically reduced because of preoperative sparing and postoperative decline in fitness due to pain or reduced mobility. This is especially the case in elderly people. Physical exercise is also an effective way to improve the fitness of such patients. Patients often do not know which exercises can be performed without overloading an implant or bone and consequently endangering surgical success (e.g., implant subsidence). Cycling is one of the most popular sports, along with swimming, aerobic exercise and jogging [1]. Patients with low back pain and those who have undergone spinal surgery are often unsure whether exercising on a bicycle ergometer will interfere with their recovery. In contrast to jogging, cycling does not lead to impact loading of the spine; however, there have been few experimental studies of the actual spinal loads during cycling [2] and how they compare to those during walking.

Direct measurement of the complete spinal load is currently not possible. However, there are a variety of ways to indirectly measure spinal loads. Intradiscal pressure has been measured *in vivo* for many activities [3]–[5], but no data for cycling are available. Another way to quantify spinal loads is to measure the induced change in spinal length, termed ‘spinal shrinkage’. Measurements indicate that after 1 h of cycling at a constant speed of 12 km/h, the shrinkage is only half of that after 1 h of erect standing [2]. However, this method of measurement provides only relative values and does not predict the real spinal forces acting on an implant during different activities.

A severe fracture of a vertebral body and tumors of the spine are often surgically treated by implantation of a vertebral body replacement (VBR) [6]. The vertebral body and the adjacent intervertebral discs are at least partially removed and replaced with the metallic implant. Several studies have used a telemeterized VBR to investigate the forces and moments on the implant during different activities [7]–[12]. However, no data were reported for cycling. Knowledge about VBR loads will be useful for the validation of computer models created to predict spinal loads.

Therefore, the aim of the current study was to document the effect of power level on the forces on a VBR during ergometer cycling.

## Materials and Methods

### Ethics Statement

The Ethics Committee of Charité – Universitätsmedizin Berlin approved the clinical implantation of the modified VBR in patients and subsequent measurements (registry number: 213-01/225-20). The procedure was explained to the patients prior to surgery, and they gave their written consent to the implantation of an instrumented VBR, the subsequent measurements and publication of their images. Measurements were permitted within a maximum period of 6 years.

### Telemeterized VBR

To measure implant loads, the clinically used VBR Synex (Sythes Inc., Bettlach, Switzerland) was modified. Six strain gauges, a 9-channel telemetry unit and a coil for the inductive power supply were integrated within a hermetically closed cylindrical tube. Endplates of various heights that were attached using screws allowed for intraoperative adaptation of the implant height to the defect dimensions. After extensive calibration by applying 21 different load combinations, the implant was used to measure the 3 force and 3 moment components acting on it. The average errors were lower than 2% for the force and 5% for the moment components relative to the maximum applied force (3,000 N) and moment (20 Nm), respectively. The sensitivity of the implant was less than 1 N for the forces and 0.01 Nm for the moments. The telemetry was only active within a magnetic field of 4 kHz. The implant and the measurement accuracy have been described in detail elsewhere [13].

### Patients

Within a period of more than 2 years, only 5 patients were found who required surgical stabilization of their spine and were qualified for a telemeterized VBR. The patients (WP1 to WP5) were suffering from an A3 type compression fracture [14] of a lumbar vertebral body. In four patients, the vertebral body L1 was fractured, and in one patient (WP5), L3 was fractured. The fractures were first stabilized from the posterior using an internal fixation device. In a second surgery, parts of the fractured vertebral body and the adjacent intervertebral discs were removed, and the instrumented VBR was inserted into the corpectomy defect. Autologous bone material was added to enhance interbody fusion. At the time of surgery, the patients were between 62 and 71 years of age. More information about the patients and the surgical procedure is provided in Table 1.

**Table 1:** Data on the patients and surgical procedures.

Parameter	Patient			
	WP1	WP2	WP4	WP5
Age at the time of surgery (years)	62	71	63	66
Height (cm)	168	169	170	180
Body mass (kg)	66	74	60	63
Fractured vertebra	L1	L1	L1	L3
Level of the internal fixation device	T12-L2	T12-L2	T11-L3	L2-L4
Time between implantation and measurement session (months)	65	13	49	15

### Measurements

For the load measurements, a coil was placed around the patient’s trunk at the level of the implant, and an antenna was secured on the patient’s back. The coil and the antenna did not restrain the patient during the exercises. During the measurements, the patient’s images were synchronously recorded on a digital videocassette with the load-dependent telemetry signals [15]. This allowed for later analysis of implant loads and motions without the patient being present. The signals were also transferred to a notebook where the forces and moments were calculated and displayed online on a monitor.

The main aim of the study of the telemeterized VBRs was to measure the loads on the implant for a wide variety of activities under daily living conditions. Thus, in 97 measuring sessions within a period of 65 months, the loads were measured for approximately 1,000 activity and parameter combinations. Approximately 25 activities, such as standing, walking, bending (forward, backward, and lateral) of the upper body while sitting and standing, were evaluated several times during almost every measuring session. Other activities, such as whole body vibration, walking on a treadmill, and cycling on an ergometer, were performed only once. Keep in mind that the patients were over 60 years old at the time of surgery, were involved in several other load measurement studies, and were not paid for the measurements. Therefore, the number of repetitions and the time-demanding measurement of additional parameters were very limited.

Four of the 5 patients agreed to cycle during one session on an ergometer. Patient WP3 did not accept our invitation because she did not feel strong enough for this exercise. The ergometer session occurred between 13 and 65 months after surgery, depending on the availability of the patients. All 4 patients were male, felt fit on that day of the session and reported no pain.

### Exercises

The patients sat upright on the bicycle ergometer with their hands on the handlebar and attempted to maintain a cadence of 40 rpm. The pedal resistance was initially 25 W and was automatically increased every 60 sec to the next power level up to 95 W. The power levels were 35, 50, 60, 70 or 75, and 85 W. Two patients were only able to cycle up to 85 W. The bicycle ergometer had a crank length of 17.5 cm. The height of the saddle was adjusted to the patient’s leg length. No straps were used on the pedals.

### Evaluation

The resultant force acting on the VBR is presented here as the geometric sum of the three measured force components. The maximum force during a single pedal revolution is the ‘peak force’, and the difference between the maximum and minimum force magnitude is the ‘force range’. For each power level, the medians of the peak forces and the force ranges were determined from an average of 20 pedal revolutions. The values obtained during cycling at 85 W were compared with the values during level walking and relaxed standing that were measured on the same day. The cycling values were also compared to those during the lifting of a 10 kg weight from the ground. Walking is an important regular daily activity with high spinal loads. Relaxed standing is one of the best reproducible positions and was measured an average of 9 times during each measurement session. Lifting a weight from the ground is the activity that caused the highest forces on the VBR.

Only descriptive statistics could be applied because no more than 4 patients with a telemeterized VBR could be included in the study and not all of them cycled at all power levels.

During cycling, the power generated is the product of the angular velocity and the crank torque (the crank length multiplied by the average mostly vertical pedal force during a revolution). The ergometer displays the power and the cadence (cadence multiplied by 2×Pi delivers the angular velocity), while the crank length is constant and known. With these data, the average pedal force during a pedal revolution can be calculated. For the various power levels, the average pedal forces were estimated and compared with the corresponding force ranges on the VBR. It was assumed that the changes in the forces of those muscles that span the hip region correlate with the average pedal force and with the force range on the VBR.

## Results

**Figure 1:**
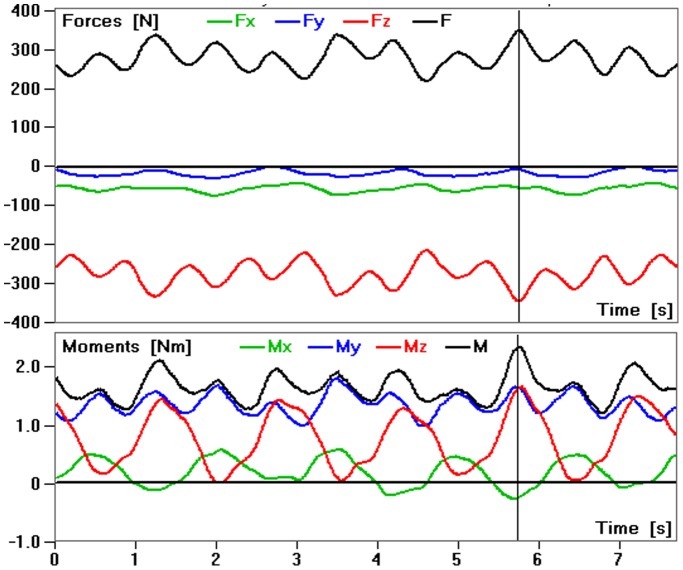
Measured loads. Force and moment components and the resultant values during cycling on an ergometer at approximately 40 rpm and a power level of 85 W. The loading curves for 5 pedal revolutions of patient WP1 are shown.

Figure 1 shows a typical example of the measured components and the resultant loads on the VBR for 5 successive randomly chosen pedal revolutions at a cadence of approximately 40 rpm and a power of 85 W. The peak values and the force ranges varied considerably.

**Figure 2:**
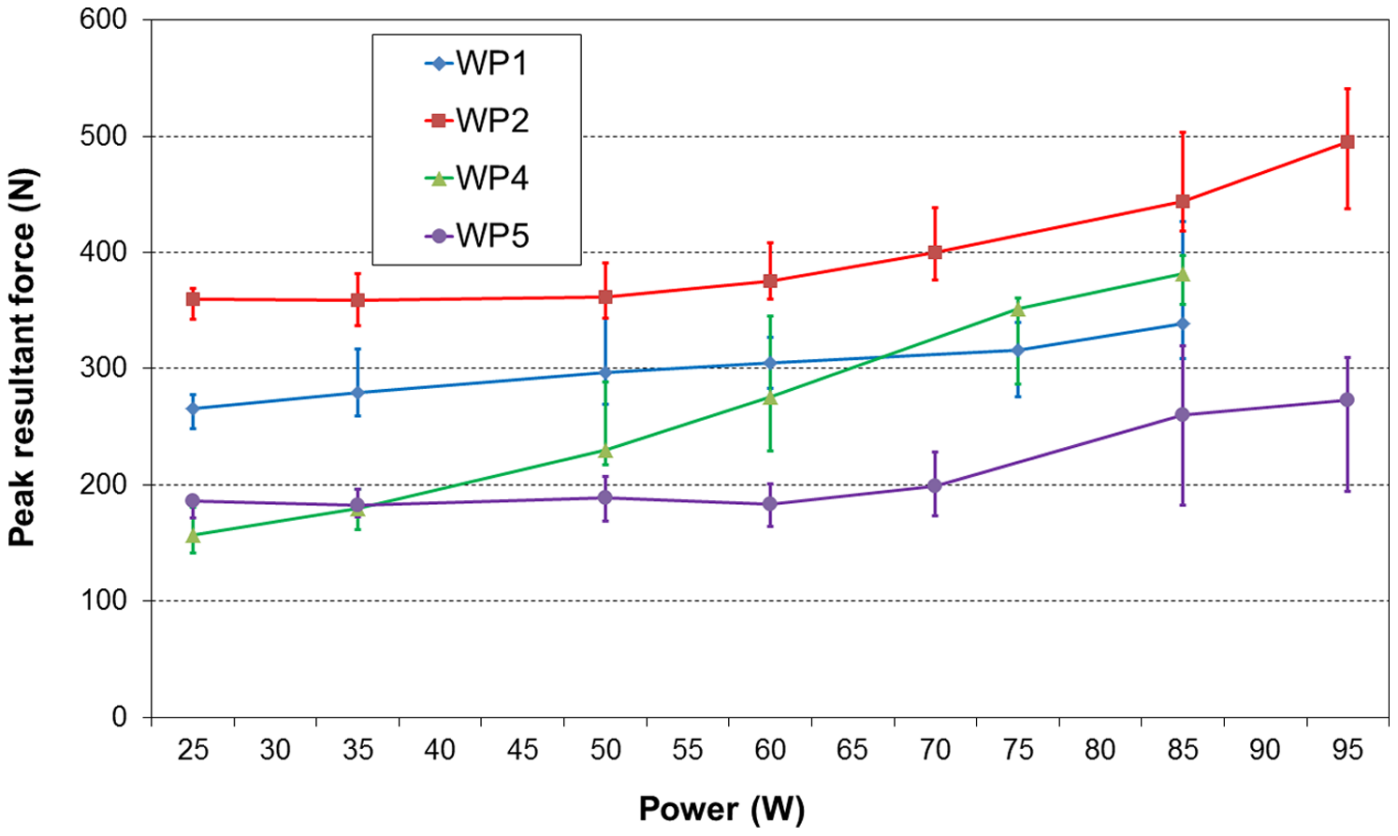
Peak resultant force versus power. The influence of the power level on the peak resultant force on the implant during cycling. The medians and ranges are shown for various power levels for the four patients (WP1, WP2, WP4 and WP5).

The peak resultant force on the VBR generally increased as the cycling power was increased (Figure 2). However, the increase varied for the different patients. For patients WP1 and WP4, there was a nearly constant force increase; the increase was progressive for patient WP2; and no increase was observed below 60 W for patient WP5. The average force increases between 25 and 85 W were 73, 84, 225 and 75 N for patients WP1, WP2, WP4 and WP5, respectively. The peak force usually occurred in the first half of the downward motion of the pedal. For two patients (WP1 and WP2), the median of the peak values for cycling was lower than that for walking measured on the same day, but for the other two patients (WP4 and WP5), the value for cycling was higher (Table 2) [8]. The peak value for cycling was higher than the average value for standing for all 4 patients [9]. By comparison, the maximum force on the VBR measured when patient WP4 lifted a weight of 10 kg from the ground was 1650 N.

**Table 2:** Comparison of the average peak force values (in N) for cycling at a power of 85 W, level walking [8] and relaxed standing [9] measured on the same day and for lifting of a 10 kg weight from the ground.

Patient	Cycling	Walking	Standing	Lifting 10 kg
WP1	339 (308–426)	422 (352–464)	318 (294–347)	944 (578–1230)
WP2	444 (419–503)	578 (520–674)	320 (288–351)	1225 (1050–1452)
WP4	381 (356–397)	365 (325–430)	210 (196–227)	1380 (1131–1649)
WP5	260 (183–320)	166 (139–232)	92 (80–114)	1129 (732–1361)

The values in parenthesis represent the ranges.

**Figure 3:**
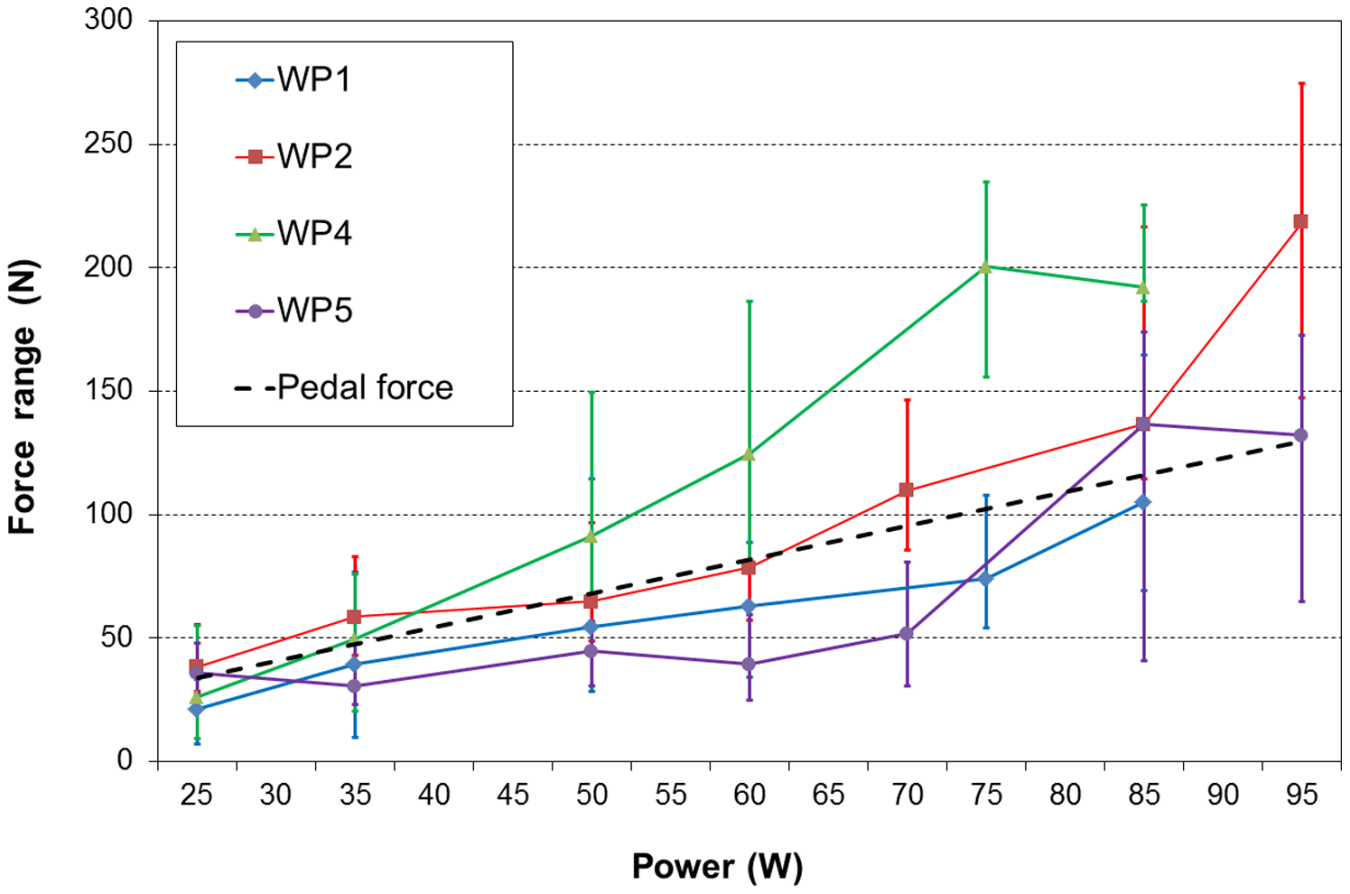
Force range versus power. The influence of the power level during cycling on the force ranges during a pedal revolution. The medians and ranges are shown for various power levels for the four patients (WP1, WP2, WP4 and WP5). The calculated average pedal force is represented by the dashed line.

Similar to the peak force, the force range during a pedal revolution also generally increased with increasing cycling power (Figure 3). The average range increases between the power levels 25 and 85 W were 84, 98, 166 and 101 N for patients WP1, WP2, WP4 and WP5, respectively. There were large intra- (Figure 1) and inter-individual (Figures 2 and 3) variations in the measured peak forces and the force ranges during a revolution. The magnitudes of the calculated average pedal force for the various power levels were similar to those of the average VBR force ranges during a pedal revolution of the four patients (Figure 3).

## Discussion

The loads on a VBR during cycling at various power levels were measured in four patients. The peak force and the force range during a pedal revolution increased with increasing cycling power. There were large intra- and inter-individual variations in the measured forces.

One limitation of the study is that these unique measurements were performed in a small cohort of only four patients. The forces were measured within one session, but the values for an average of 20 revolutions were evaluated for each power level. Thus, the median value should be representative. However, repeating the measurements on a different day may lead to slightly different values due to small differences in the overall muscle tone, the orientation of the upper body or the position of the hands on the handlebar. In 2 patients the power level 70 W instead of 75 W was chosen. The higher power levels were chosen depending on the patient’s behavior during the exercise in order not to overstress them. Only the cadence of 40 rpm was studied to avoid overstressing the patients, who were involved in many other load measurement studies [7]–[12]. Measurements of telemeterized knee joints showed that higher cadences lead to smaller forces in the knee joint [16]. We expected the same trend for spinal loads. During the measurements, the patients sat upright on the ergometer with their hands on the handlebar. This may affect the magnitude of the measured force; however, this and the other limitations should not affect the general trend of the results.

Three of the VBRs were implanted at level L1 and one (in WP5) at level L3. The spinal loads at the lower level should be higher because a greater part of the upper body weight is acting there. However, the peak force on the VBR and the measured forces for other activities were mostly lower in WP5 than in the other patients. Thus, other factors such as the percentage of load taken over by the internal fixators and possible implant subsidence have a stronger influence on the VBR peak force.

Measurement of the loads during cycling was performed between 13 and 65 months after surgery. At the time of measurement, the muscle activation pattern should have normalized, and all patients felt fit. All measurements of a patient presented here were acquired in one day, except weight lifting. Thus, these measurements do not provide information about the influence of the postoperative time on the results.

The maximum spinal load during an activity varies strongly [8], [11], [17], [18]. Even for the reproducible position ‘relaxed standing’, the force on the VBR varied on average by approximately ±50 N when measured 10 times within 1 h [9]. Similar variations were found in intradiscal pressure measurements [4] and in a previous study on internal spinal fixation devices [19]. The spinal loads obviously depend on several factors such as small variations in the posture, muscle co-contractions and psychic stress, which are all difficult to control.

The pedal forces during cycling are much lower than the reaction on the foot during walking although the forces on the VBR were similar. This demonstrates that for the spinal load, muscle forces are more relevant than external forces. With increasing cycling power, the forces in the muscles that span the hip region increase. Higher trunk muscle forces lead to higher spinal forces. The trunk muscle forces also depend strongly on the location of the center of mass (CoM) of the upper body [12], [20]. An anterior shift of the CoM requires higher back muscle force to keep a stable position, which in turn leads to higher spinal forces. The position of the CoM of the upper body in relation to the spine has the strongest effect on the spinal loads.

The peak forces during cycling on an ergometer at a power of 85 W were higher than the maximum values for walking measured on the same day in 2 patients. The exact load which places a considerable risk on the spine is unknown. Walking is considered to be the most important regularly performed activity in daily life. If patients are allowed to walk then all activities with lower maximum forces than during walking should be allowed. But, to be safe, people with back problems or previous spinal surgery should not exercise on a bicycle ergometer at high power levels. However, in general, our results suggest that cycling is a suitable activity for people with back problems. For the first time, loads acting on a spinal implant were directly measured *in vivo* during cycling. These data may also be used for the validation of computer models for estimating spinal loads.
